# Image-Based Treatment Planning of the Post-Lumpectomy Breast Utilizing CT and 3TMRI

**DOI:** 10.4061/2011/246265

**Published:** 2011-04-06

**Authors:** Geraldine Jacobson, Gideon Zamba, Vicki Betts, M. Muruganandham, Joni Buechler-Price

**Affiliations:** ^1^Department of Radiation Oncology, University of Iowa Hospital and Clinics, 200 Hawkins Drive, Iowa City, IA 52242, USA; ^2^Department of Biostatistics, College of Public Health, The University of Iowa, 200 Hawkins Drive, Iowa City, IA 52242, USA

## Abstract

Accurate lumpectomy cavity definition is critical in breast treatment planning. We compared contouring lumpectomy cavity volume and cavity visualization score (CVS) with CT versus 3T MRI. 29 patients were imaged with CT and 3T MRI. Seven additional boost planning sets were obtained for 36 image sets total. Three observers contoured the lumpectomy cavity on all images, assigning a cavity visualization score (CVS ) of 1 to 5. Measures of consistency and agreement for CT volumes were 98.84% and 98.62%, for T1 MRI were 95.65% and 95.55%, and for T2 MRI were 97.63% and 97.71%. The mean CT, T1 MRI, and T2 MRI CVS scores were 3.28, 3.38, and 4.32, respectively. There was a highly significant difference between CT and T2 scores (*P* < .00001) and between T1 and T2 scores (*P* < .00001). Interobserver consistency and agreement regarding volumes were high for all three modalities with T2 MRI CVS the highest. MRI may contribute to target definition in selected patients.

## 1. Introduction

Definition of the lumpectomy cavity is a critical step in treatment planning for irradiation of the intact breast, breast boost, and for partial breast irradiation. Multiple studies have shown the limitations of single modality imaging with interobserver differences in lumpectomy cavity definition [1–4]. CT-based imaging is commonly used for breast treatment planning; but the limited soft tissue contrast of CT can result in poor visualization of the lumpectomy site in patients with dense breast parenchyma, small lumpectomy cavities, or a prolonged delay between surgery and treatment planning [2, 3]. MR imaging provides superior soft tissue contrast and may provide clearer visualization of the lumpectomy cavity. Although the diagnostic role of MRI in breast cancer management is expanding, MRI is rarely used as an imaging modality in post-lumpectomy radiation therapy planning. We compared contouring of the lumpectomy cavity volume and cavity visualization score (CVS) based on CT imaging compared to 3 Tesla magnetic resonance imaging, (3T MRI).

## 2. Methods and Materials

This is an IRB-approved retrospective review of treatment planning imaging obtained for breast cancer patients following breast conserving surgery. 

From September 2008 to July 2009, 29 patients referred for intact breast irradiation had breast imaging performed using both CT and noncontrast 3T MRI. Of these, seven patients had repeat CT and MRI performed at the time of boost planning, providing 36 image sets. Sixteen patients did not receive chemotherapy. The average interval between surgery and image acquisition for this group was 28 days (range 14–52). 

Eleven patients received postoperative adjuvant chemotherapy prior to radiation. The average interval between surgery and image acquisition was 137 days (range 68–206) for this group.

Two patients had neoadjuvant chemotherapy followed by surgery, then radiation. The surgery-image intervals of these 2 patients were 38 and 57 days.

All 36 image sets included a CT and T1 MRI. 28 image sets also included a T2 MRI. CT scans were performed with patients in the supine treatment position, both arms extended above the head on a commercial arm board, with wires defining the breast and scars. A noncontrast MRI was performed immediately afterward in the same position. MRI scans were obtained with a 3T Scanner (Siemens TRIO TIM) using a flexible six-element body RF matrix coil. The coil was placed over the patient's chest in the supine position. The 3D T1 weighted images were acquired using VIBE (Volumetric interpolated breath-hold exam) sequence with TR/TE: 3.37/1.23 ms, 1 NEX at 2.4 × 2.0 × 2.0 mm spatial resolution in parallel imaging mode (acceleration factor of 2) yielding 104 slices in 0.21 minutes. T2 weighted images were obtained using a 2D Turbo spin echo sequence with TR/TE: 6440/127 ms, 3 NEX at 2.1 × 1.6 × 3.0 mm resolution with acceleration factor of 2 yielding 30–56 slices in 6.02 minutes. Image distortions resulting from gradient field nonlinearity were corrected using a vendor supplied 3D distortion algorithm, which compensates for slice curving effects in addition to in-plane distortions. The distortion corrected images were imported into the treatment planning system (TPS). 

The MR images, as they do not contain the intrinsic electron density information of tissues required for radiation therapy planning, were registered/fused with CT images in a two-step process. First, a coarse registration was achieved using a manual interactive registration tool available with the TPS (Pinnacle^3^ RTP, Phillips Medical Systems (Cleveland), Inc., Fitchburg, WI, USA), which permits rigid-body transformations (translations and rotations) on the secondary image set (MRI) along and about the three major axes of the primary (CT). Second, an automatic Local Correlation (LC) registration algorithm (available with Syntegra registration module of Pinnacle^3^ RTP) was applied to further enhance the accuracy. The visualization tools-sliding window and/or checkerboard were used to verify the clinical accuracy of the fusion process.

Three observers, a radiation oncologist, a dosimetrist, and a radiation oncology resident independently contoured the lumpectomy cavity on CT and MRI images (Table 1). Surgical clips were occasionally, but not routinely, placed in these patients by referring surgeons. When present, clips were contoured independently as clips, not as part of the lumpectomy cavity. Contourers outlined the visible seroma cavity/tumor bed on CT. Associated stranding was not contoured. For T1 and T2 MRI, the contourers outlined the outermost contour of the boundary between tumor bed and breast tissue. Measures of consistency and agreement between observers for CT volume and MRI volume were evaluated by the Intraclass Correlation Coefficient (ICC) obtained from a random effect ANOVA model. All patients had CT and T1 MRI scans; 28 had an additional T2 MRI scan. Statistics are based on comparing CT images with T1 and T2 MRI images. The observers assigned each image a cavity visualization score (CVS) of 1 (cavity not visualized) to 5 (all cavity margins clearly defined), see Table 2 (1).

## 3. Results

**Figure 1:**
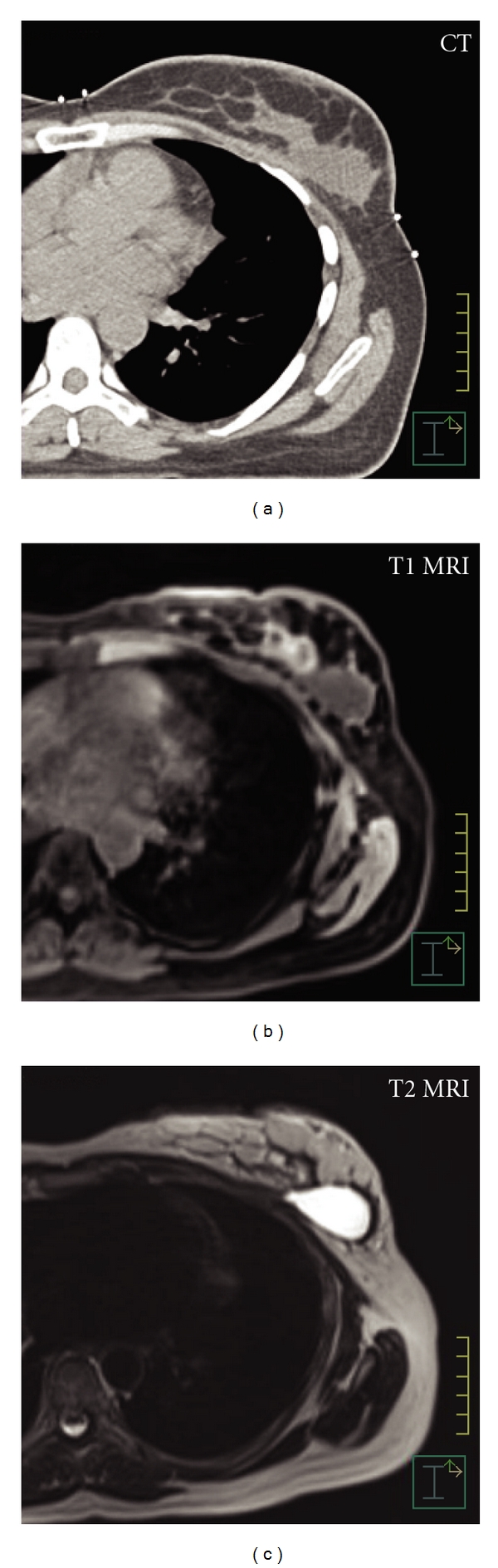
Comparison of imaging modalities. (a) CT shows homogeneous gray area. Borders are distinct laterally. Medially, the borders between lumpectomy cavity and breast parenchyma and chest wall are poorly defined. CVS 3. (b) T1 MRI, noncontrast, shows cavity with fairly well-defined borders, some rim enhancement. CVS 4. (c) T2 MRI noncontrast shows hyperintense signal consistent with seroma, distinct margins. CVS 5.

**Figure 2:**
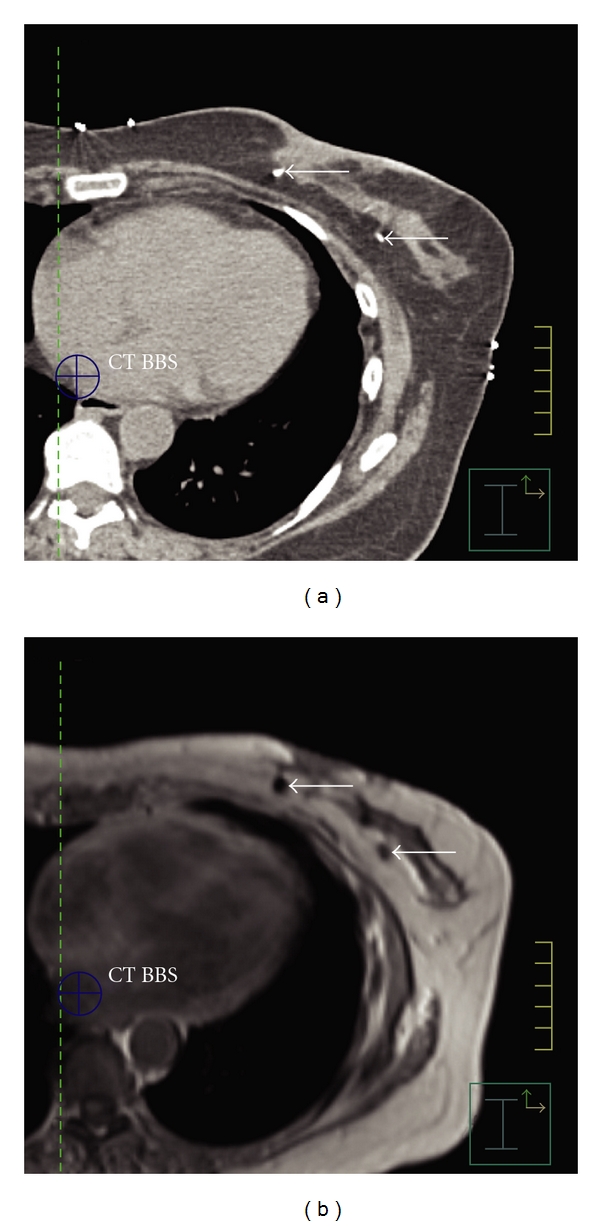
(a) Biopsy clips visible on CT. (b) Biopsy clips seen as signal void on T1 MRI.

Measures of consistency and agreement for CT volumes were 98.84% and 98.62%. Measures for T1 MRI were 95.65% for consistency and 95.55% for agreement. Measures for T2 MRI were 97.63% for consistency and 97.71% for agreement. There was a strong and significant agreement between observers. Observations do not differ much in assessment between CT and MRI. However, there was a significant difference in the perceived quality of the image measured by cavity visualization score (CVS), see Figure 1. The mean CT, T1 MRI, and T2 MRI scores were 3.28, 3.38, and 4.32, respectively. Analysis of variance (one-way ANOVA) was used to compare all three CVS at once. There was a significant difference between the scores (*P* value <  .0001). Pairwise comparisons showed no significant difference between CT scores and T1 scores (*P* = .43). There was a highly significant difference between CT and T2 scores (*P* < .00001) and between T1 and T2 scores (*P* < .00001). Surgical clips, when present, are easily seen on CT. They can sometimes be visualized on T1 MRI by signal void, as seen in Figure 2.

## 4. Discussion

Breast conservation therapy with tumor directed surgery followed by whole breast radiation has been an accepted management of invasive breast cancer for several decades. Recent trends in radiation oncology have focused on image-based planning for the majority of disease sites including breast cancer. The first step in image-based treatment planning of the intact breast following breast conserving surgery is accurate identification and contouring of the target volume. Most series related to boost definition and partial breast radiation therapy have defined the post-lumpectomy site as the volume on which subsequent planning is based. Numerous studies have demonstrated that there is a great deal of variability and uncertainty in this critical first step. The ability to identify the lumpectomy cavity varies from patient to patient. Several factors, including breast density, interval between surgery and image acquisition, and the volume of the lumpectomy cavity, can present challenges in distinguishing the lumpectomy site from the normal breast with CT images. In a few instances, particularly when several cycles of chemotherapy are delivered between lumpectomy and initiation of radiation, the site of the lumpectomy cavity cannot be identified. Even when visible, postoperative stranding, borders between the lumpectomy site and chest wall or skin, and dense breast parenchyma can obscure the borders of the lumpectomy cavity. In addition to the challenges in identification of the lumpectomy site, several studies have shown interobserver differences in contouring radiation target volumes [5–7]. Rates of discordance in interobserver studies have conformity indices approaching 50% [2, 8]. An RTOG multi-institutional and multiobserver study showed clinically significant differences in target and organ at risk delineation for breast irradiation, with some structure overlaps as low as 10% and volume variations with standard deviations as high as 60% [9].

MRI has improved soft tissue characterization compared to CT and is used in breast cancer screening and presurgical evaluation. As a screening modality, breast MRI has been found to have a sensitivity of 93–100% [10, 11]. In a single institutional retrospective study, presurgery breast MRI changed breast cancer surgical management in 9.7% of newly diagnosed cases [12]. Although MRI has a high resolution and sensitivity in breast tissue, it has not been widely used in radiation therapy treatment planning. Reasons may include lack of access, cost, and lack of data on the utility of MRI imaging for this purpose. In addition, there are issues related to patient position, soft tissue deformation, and spatial accuracy. Ahn et al. designed an MRI imaging protocol for treatment planning with the patient in a prone position and demonstrated adequate contrast and spatial fidelity [13]. Whipp and Halliwell obtained postoperative MRIs in 100 randomly selected breast cancer patients [14]. The images were obtained in a single open MRI scanner using the conventional breast radiotherapy treatment position, without contrast, prior to routine two-dimensional simulation. MRI results were qualitatively different from CT and ultrasound cavities described in the literature. 85% of the MRI volumes were described as heterogeneous, 9% were described as homogeneous. 88% were described as complex/cystic and 5% as simple cystic. Regular concentric rings of differing signal were seen within the postoperative complex in 32% of scans. The postoperative complex was in contact with the chest wall in 53% of patients. A follow-up study by this group reported on local recurrence rates after MRI-assisted radiotherapy planning [15]. The lumpectomy site, described as the postoperative complex (POCx) was visible on MRI of all 221 patients. MRI imaging was used in the context of conventional treatment planning and altered the standard field margins in 69% of patients. 

The University of Iowa Hospital and Clinics Department of Radiation Oncology has a 3T MRI device present in the department for treatment planning. Although diagnostic breast MRI is usually obtained in a prone position, we preferred to continue our treatment planning and treatment delivery in the supine position. Many of our patients have a BMI greater than 30 and would be uncomfortable in a prolonged prone position. Image acquisition at 3T using surface coils combined with the parallel imaging approach resulted in an improved SNR and adequate coverage. Since the MRI was obtained for treatment planning and not for diagnosis, we did not use contrast. We used a surface coil and found minimal deformation of breast tissue. Obtaining the noncontrast T1 and T2 images adds an additional 16 minutes to the treatment planning time. As described above, the MRI images provided a greater detail than the CT images, showing heterogeneous cavities and concentric rings of granulation tissue. The lumpectomy cavity is identified by a bright signal on the T2 image and shows clear demarcation between seroma and normal tissue. In this study, the T2 images provided the best cavity visualization score, which was significantly better than that of the CT or T1 MRI. MRI, particularly T2 MRI, better demarcated the interface between seroma and chest wall, seroma and skin, and distinguished between seroma and dense breast parenchyma (Figure 1).

## 5. Conclusion

MRI provides more detailed visual information than CT in the post-lumpectomy breast. In patients with difficult to visualize cavities, the addition of MRI images to CT treatment planning may contribute to improved target volume definition. In our experience, a noncontrast MRI image can be obtained in the supine treatment planning position in a reasonable period of time during the treatment planning session.

##  Conflict of Interests

 The authors declare that no conflict of interest or potential conflict of interest exists.

## Figures and Tables

**Table 1:** Patient and imaging data.

	Median	Range
Age (years), *N* = 29	56.9	38–76
Interval from surgery to image acquisition (days), *N* = 29		
No chemotherapy *N* = 16	28	14–52
Adjuvant postoperative chemotherapy *N* = 11	137	38–206
Neoadjuvant chemotherapy-before surgery *N* = 2	47.5	38–57
Lumpectomy volume (cc) (Average of 3 contourers)		
CT (*n* = 36*)	43.88	4.36–239.85
T1 MRI (*n* = 36)	40.94	4.51–285.97
T2 MRI (*n* = 28)	35.18	5.02–176.76
*n = 29* patients with 7 boosts*		

**Table 2:** Cavity visualization score (CVS).

CVS	Description
1	Cavity not visualized
2	Cavity visualized but margins indistinct
3	Cavity visualized with some distinct margins
4	Cavity with majority of margins distinct
5	All margins clearly seen
